# Expected values for gastrointestinal and pancreatic hormone concentrations in healthy volunteers in the fasting and postprandial state

**DOI:** 10.1177/0004563220975658

**Published:** 2020-12-07

**Authors:** Claire L Meek, Hannah B Lewis, Keith Burling, Frank Reimann, Fiona Gribble

**Affiliations:** 1 Wellcome Trust-MRC Institute of Metabolic Science, Metabolic Research Laboratories, University of Cambridge, Addenbrooke’s Hospital, Cambridge, UK; 2 Department of Clinical Biochemistry, Cambridge University Hospitals, Addenbrooke’s Hospital, Cambridge, UK; 3 Core Biochemical Assay Laboratory, Addenbrooke’s Hospital, Hills Road, Cambridge, UK

**Keywords:** Glucagon-like peptide 1 (GLP-1), glucose-dependent insulinotropic polypeptide (GIP), peptide YY (PYY), analytical chemistry, immunoassay

## Abstract

**Background:**

Gastrointestinal hormones regulate intestinal transit, control digestion, influence appetite and promote satiety. Altered production or action of gut hormones, including glucagon-like peptide-1 (GLP-1), glucose-dependent insulinotropic polypeptide (GIP) and peptide YY (PYY), may contribute to the biological basis of obesity and altered glucose homeostasis. However, challenges in analytical methodology and lack of clarity on expected values for healthy individuals have limited progress in this field. The aim of this study was to describe expected concentrations of gastrointestinal and pancreatic hormones in healthy volunteers following a standardized meal test (SMT) or 75 g oral glucose tolerance test (OGTT).

**Methods:**

A total of 28 healthy volunteers (12 men, 16 women; mean age 31.3 years; mean body mass index 24.9 kg/m^2^) were recruited to attend a hospital clinic on two occasions. Volunteers had blood sampling in the fasting state and were given, in randomized order, an oral glucose tolerance test (OGTT) and standardized mixed liquid meal test with venepuncture at timed intervals for 4 h after ingestion. Analytical methods for gut and pancreatic hormones were assessed and optimized. Concentrations of gut and pancreatic hormones were measured and used to compile ranges of expected values.

**Results:**

Ranges of expected values were created for glucose, insulin, glucagon, GLP-1, GIP, PYY and free fatty acids in response to a standardized mixed liquid meal or OGTT. Intact proinsulin and C-peptide levels were also measured following the OGTT.

**Conclusions:**

These ranges of expected values can now be used to compare gut hormone concentrations between healthy individuals and patient groups.

## Introduction

The rising prevalence of nutritional disorders has led to increased scientific interest in the mechanisms which regulate nutrient disposal, appetite and satiety. Hormones such as glucagon-like peptide-1 (GLP-1) and peptide YY (PYY), which are released from the gastrointestinal tract in response to nutrient ingestion, may mediate satiety and reduce appetite.^1,2^ GLP-1- and glucose-dependent insulinotropic polypeptide (GIP) function as incretins, promoting the release of insulin from the pancreas in response to a meal.^3^ The complex relationships between gastrointestinal and pancreatic hormones are now thought to regulate energy balance and may be affected by diseases such as type 2 diabetes mellitus (T2DM).^4^ Bariatric surgery, which provides an effective method of long-term weight control, is associated with increased postprandial concentrations of GLP-1, PYY and improved glucose tolerance.^5,6^ However, the expected circulating concentrations of many gastrointestinal and pancreatic hormones in healthy individuals have yet to be established, yet are essential for the interpretation of altered levels seen in metabolic and gastrointestinal conditions.

Despite the high levels of interest in gut hormones, progress in this field has been limited by the development of robust analytical methods for their quantitation in plasma. Many of these peptides are present in picomolar concentrations and require highly sensitive assays, which can measure concentrations as low as 1–10 pmol/L. Highly specific assays are also required, as many peptides have important structural similarities. Glucagon and oxyntomodulin, for example, differ only at their C-termini and are indistinguishable using antibodies that target the shared peptide sequence.^7^ A number of commercially available assays have been shown to be unfit for purpose.^8,9^ However, analytical methodology and the concordance of results obtained using different assays are improving. With this advancement in methodology, it is now possible to permit comparison of plasma peptide concentrations in health and disease.

Concentrations of several gastrointestinal hormones are altered after bariatric surgery,^10^ in individuals with T2DM^4^ and after gestational diabetes.^11^ However, expected concentrations of a comprehensive range of gut hormones in healthy individuals have not been described in depth. The aim of the current study was to provide ranges of expected values using reliable and readily-available assays for gastrointestinal and pancreatic hormones in healthy human subjects in the fasting state and after both an oral glucose tolerance test (OGTT) and a standardized mixed liquid meal test (SMT).

## Participants and methods

### Study design

Healthy male and female volunteers aged 18–65 years old were recruited using advertisements placed in Addenbrooke’s Hospital and the University of Cambridge. To fulfil the entry criteria, healthy volunteers needed to be free from chronic diseases and recent acute conditions, such as infections, diarrhoea or constipation, and have a body mass index (BMI) of 18–35 kg/m^2^. Healthy participants were either taking no medication or were stable on medication that was considered unlikely to interfere with the results of the study. Participants with known pre-existing anaemia, diabetes or endocrine disorders and pregnant or lactating women were excluded from this study. The study was given ethical approval by the local research ethics committee (13/EE/0195), and all participants gave full written consent.

### Study visits and stimulation tests

Participants attended a Clinical Research Facility at 09:00 h on two occasions following an overnight fast. The evening before each visit, participants prepared for themselves a standardized pasta meal containing 15% protein, 30% fat and 55% carbohydrate which was designed to provide 33% of their daily calorie requirement based on an estimation of their metabolic rate and activity levels.^12^ After the meal, participants were allowed free access to water but were asked to avoid food, caffeinated and calorie-containing drinks overnight for 12 h prior to the study visit.

Participants attended for two visits and received a 75 g OGTT and a SMT in randomized order (Table 1 for composition). Blood was taken at baseline and at timed intervals (0, 15, 30, 45, 60, 90, 120, 150, 180, 210 and 240 min) after the OGTT or SMT for analysis of glucose, non-esterified-free fatty acids (FFA) and pancreatic and gut hormones. Participants were asked to remain sedentary throughout the testing process.

**Table 1.** Nutritional contents of OGTT and Ensure plus standardized meal.

Nutrient	Ensure Plus (standardized meal)	75 g OGTT
Volume	237 mL	250 mL
Energy content	350 kcal, 1464 kJ	300 kcal, 1255 kJ
	57% from carbohydrate, 15% from protein and 28% from fat.	100% from carbohydrate
Carbohydrates, g	50	75
Of which, sugars, g	20	75
Protein	13	0
Fat	11	0
Vitamins (25–50% recommended daily intake)	Vitamins A, B1, B2, B6, B12, C,D,E,K, biotin, pantothenic acid and folate.	0
Minerals (8–60% recommended daily intake)	Calcium, iron, phosphorus, iodine, magnesium, zinc, selenium, copper, manganese, chromium, molybdenum, chloride.	Unknown, depends on water mineral content.

OGTT: oral glucose tolerance test.

The OGTT was produced using 82.5 g glucose monohydrate (equivalent to 75 g pure glucose) which was dissolved in 250 mL chilled water, and a further glass of 250 mL water was given afterwards to wash out the oral cavity. The SMT consisted of a 237 mL bottle of Ensure plus, a balanced nutritional supplement containing 11 g fat (28%), 13 g protein (15%) and 50 g carbohydrate (57%). Participants were also given 250 mL of water after the meal was consumed. Each drink (OGTT or SMT) was consumed within a 5 min time frame. Altogether, the OGTT and SMT provided 300 and 350 kcals, respectively.

### Analytical methodology

All analyses were performed in the Core Biochemical Assay Laboratory in Addenbrooke’s Hospital. The analytical methods used with performance measures are given in Table 2 (see supplemental information for more details). All analyses demonstrated acceptable linearity and recovery and passed standard quality control measures. Samples with known common interferences such as haemolysis, lipaemia and hyperbilirubinaemia were excluded from this analysis.

**Table 2.** Summary of methods used for analysis of biochemical markers, gut and pancreatic hormones.

Analyte	Matrix	Specimen preparation	Instrument/kit	Method	Typical intra-assay CVs	Range
Glucose	Lithium heparin Plasma	Centrifuged at 3500 *g* at 4°C for 10 min. Rapidly frozen.	Siemens’ Dimension	Hexokinase method	<2%	0.1–27.8 mmol/L
Insulin	Lithium heparin plasma	Placed immediately on ice. Centrifuged at 3500 *g* at 4°C for 10 min. Rapidly frozen.	Diasorin Liaison	Sandwich immunoassay with chemilumine-scent detection	of 5.0–6.0%	3–3000 pmol/L
GLP-1	EDTA plasma	Placed immediately on ice. Centrifuged at 3500 g at 4°C for 10 min. Rapidly frozen.	Mesoscale discovery total GLP-1 kit	Sandwich immunoassay with electro-chemilumine-scent detection	5.2–8.2%; 15.4% near LLOD.	1.4–1000 pg/mL
PYY	Clotted serum	Clotted for 10 min at room temperature. Centrifuged at 3500 *g* at 4°C for 10 min. Rapidly frozen.	Mesoscale Discovery total PYY kit - includes PYY (1–36) and PYY (3–36)	Immunoassay	7.8–16.4%	30–3000 pg/mL
GIP	EDTA plasma	Placed immediately on ice. Centrifuged at 3500 *g* at 4°C for 10 min. Rapidly frozen.	Mesoscale Discovery Total GIP kit	Immunoassay	9.3–11.0%	1.0–2500 pg/mL
FFA	Clotted serum	Clotted for 10 min at room temperature. Centrifuged at 3500 *g* at 4°C for 10 min. Rapidly frozen.	Roche FFA kit	Enzymatic colorimetric assay	5.0–12.3%	50–1500 μmol/L
Glucagon	EDTA plasma	Placed immediately on ice. Centrifuged at 3500 *g* at 4°C for 10 min. Rapidly frozen.	Mercodia Glucagon kit	Sandwich immunoassay	8–10%; 21.2% at LLOD	5–1000 pmol/L
C-peptide	Lithium heparin plasma	Placed immediately on ice. Centrifuged at 3500 *g* at 4°C for 10 min. Rapidly frozen.	Diasorin Liaison	Sandwich immunoassay with chemiluminescent detection	4.6–7.6 %	9–9900 pmol/L
Proinsulin	Lithium heparin plasma	Placed immediately on ice. Centrifuged at 3500 *g* at 4°C for 10 min. Rapidly frozen.	Auto-DELFIA	Fluorimetric immunoassay	4.9–11.8%	1.25–100 pmol/L

CV: coefficient of variation; EDTA: ethylenediaminetetraacetic acid; FFA: free fatty acids; GLP-1: glucagon-like peptide-1; GIP: glucose-dependent insulinotropic polypeptide; PYY: peptide YY.

### Statistical analysis

Characteristics of participants are described as mean (±SD). A range of expected values was produced using the non-parametric method as recommended by the IFCC through the Clinical and Laboratory Standards Institute (CLSI).^13^ The 2.5th and 97.5th percentiles were calculated to give an interval which included 95% of values from this sample. Other approaches, such as bootstrapping and robust methods were not used in this study but may have some advantages in a small sample.^14^

The appropriate assessment of outliers is important in establishing ranges of expected values. Some outliers were identified and were assessed for each individual participant and each analyte. If any clear pathological cause was evident, the outlier was removed. However, in practice, the outliers could not be explained on the basis of known pathology and were not excluded. Some individuals were found to have elevated fasting glucose and insulin concentrations. Although these findings are suggestive of prediabetes, they are also extremely common in the reference population, and these data were therefore kept in the analysis.

## Results

A total of 28 healthy volunteers were recruited (12 male, 16 female; Table 3). Most healthy volunteers were aged 22–40 years (31.3 ± 10.9 years) with a lean body mass index 24.9 ± 3.7 kg/m^2^. Participants had normal haemoglobin, white cell count, alanine aminotransferase (ALT), thyroid-stimulating hormone (TSH) and creatinine at baseline with an HbA1c in the non-diabetic range (33.6 ± 3.7 mmol/L).

**Table 3.** Baseline characteristics of study participants. Characteristics shown as mean (SD) for continuous variables and *n* (%) for categorical variables.

Characteristic	Mean (SD)
Sex, male	12/28 (42.9%)
Age (years at enrolment)	31.3 (10.9)
Body mass index kg/m^2^	24.9 (3.7)
Haemoglobin g/L	135 (12)
White cell count ×10^9^/L	6.1 (1.6)
Platelets ×10^9^/L	223 (47)
Creatinine μmol/L	80.1 (14.7)
ALT IU/L	30.6 (9.8)
TSH U/L	1.6 (0.6)
HbA1c mmol/mol	33.6 (3.7)

**Figure 1.**
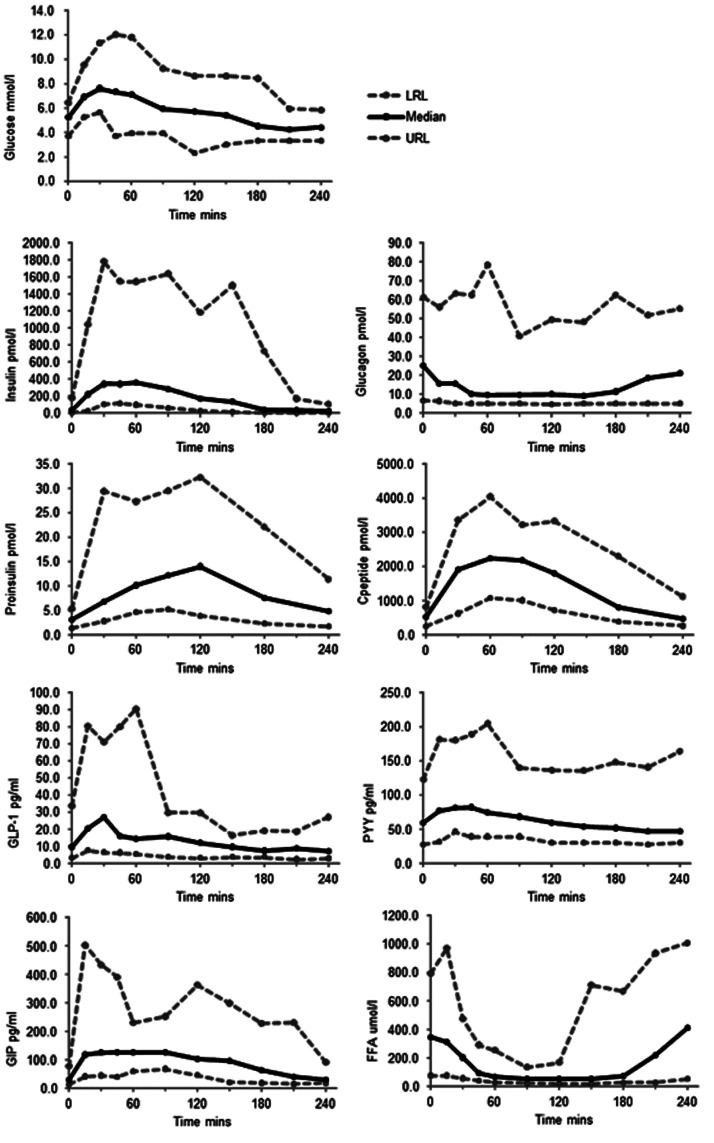
Ranges of expected values for glucose, insulin, glucagon, intact proinsulin, C-peptide, GLP-1, PYY, GIP and FFA in the fasting state and after a 75 g oral glucose tolerance test (OGTT). Note that intact proinsulin and C-peptide were measured at time-points 0, 30, 60, 90, 120, 180 and 240 minutes only on samples from 20 participants. The remaining analytes were measured on samples from 28 participants. FFA: free fatty acids; GLP-1: glucagon-like peptide-1; GIP: glucose-dependent insulinotropic polypeptide; LRL: lower range limit; 2.5th percentile; PYY: peptide YY; URL: upper range limit; 97.5th percentile.

**Figure 2.**
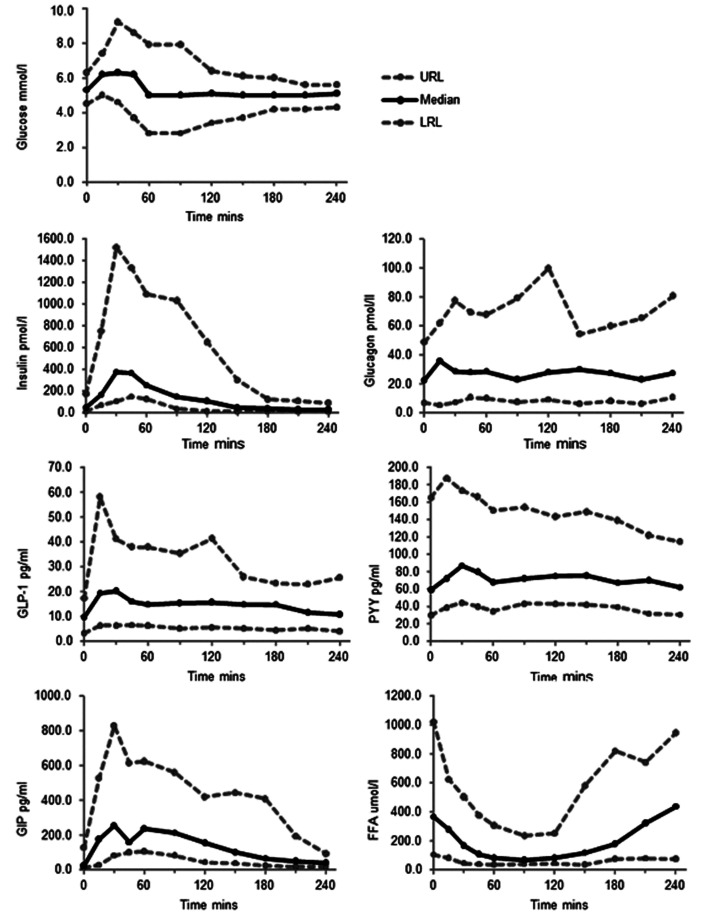
Ranges of expected values for glucose, insulin, glucagon, GLP-1, PYY, GIP and FFA in the fasting state and after a standardized meal test (SMT), based on measurements taken from 28 participants. FFA: free fatty acids; GLP-1: glucagon-like peptide-1; GIP: glucose-dependent insulinotropic polypeptide; LRL: lower range limit; 2.5th percentile; PYY: peptide YY; URL: upper range limit; 97.5th percentile.

Data from these 28 participants were used to create ranges of expected values for glucose, insulin, glucagon, total GLP-1, PYY, GIP and FFA before and after an OGTT and SMT (see Figures 1 and 2; Tables S1 and S2). C-peptide and intact proinsulin were measured following an OGTT only (*n* = 20 participants; Figure 2).

## Discussion

In this study, ranges of expected values were provided for glucose, insulin, glucagon, total GLP-1, PYY, GIP and FFA in healthy volunteers in fasting and postprandial circumstances, in response to both an OGTT and a standardized mixed liquid meal. Ranges for intact proinsulin and C-peptide were produced in healthy volunteers following an OGTT. These ranges of expected values provide reference values for comparison with concentrations in various disease states in future studies. There is currently no standardized stimulation test for the assessment of postprandial gut hormone concentrations. Previous studies have used a variety of stimuli including standardized breakfasts, mixed liquid meals, glucose and ice cream,^15–19^ which presents considerable challenges in the comparison of findings. As gut hormone release is triggered by protein and fat, as well as glucose, a mixed meal is generally a well-tolerated and reliable stimulus of gut hormone secretion. The liquid standardized meal used in the current study circumvents the requirement for mechanical food breakdown in the stomach, and is therefore suitable for comparison with patient groups, such as postbariatric or other gastrointestinal surgery. Oral glucose tolerance tests using a 75 g glucose bolus are frequently used for the assessment of glucose metabolism, but are not well tolerated after gastric bypass surgery.

To assess variability in gut hormone secretion, our data suggest that sampling at 0 and 30 min time-points would encompass the majority of variability in hormone measurements in healthy subjects, although a longer period of sampling may be required in populations where gastric emptying is more variable and in response to solid meal stimuli. At later time points, the inhibitory effects of elevated GLP-1 and PYY concentrations on gastric emptying tend to reduce the rate of nutrient delivery into the small intestine. This means the gut hormone concentrations then become a complex balance between the strength of the feedback control of gastric emptying, the quantity of nutrients still awaiting digestion and absorption and the secretory potential of the small intestine.

Gut hormone analysis was based on methods that are routine within our laboratory and we have found to be reliable in most circumstances. Detailed methodology and performance data have been given for each analyte (Table 2 and supplemental material). Many other studies advocate the use of protease inhibitors and DPP-IV inhibitors which can be added to blood tubes prior to sampling or provided in the form of specialized pretreated tubes.^20^ DPP4 or protease inhibitor-treated tubes provide results up to 20–30% higher for some analytes,^21^ which reduces the need for high analytical sensitivity and may increase analytical performance. However, in this study, we chose specimen preparation and processing techniques that minimize degradation while using equipment and blood tubes readily available in most health-care environments and require no added reagents. Although rapid processing is essential for gut hormone and insulin analyses, these sample preparation methods were found in this study to be feasible both in the hospital research unit and have been tested in a mobile laboratory in the field. DPP4 inhibitors would be required for the measurement of active GLP-1 concentrations, but in the current study, we elected to assay total GLP-1 as a better potential measure of the hormone secretory rate.

Choosing a suitable group of individuals in perfect health for the development of ranges of expected values is challenging. The study group used here were relatively young and lean and were recruited using advertisements in University and Hospital buildings. Although most participants took no regular medication, several of the female participants were on the oral contraceptive pill, and this may have influenced the results. Gastrointestinal and pancreatic hormone concentrations were not studied at different specified times during the female menstrual cycle or in post-menopausal women. Our study group were predominantly European and ethnic differences in GLP-1 responses have been previously identified.^22^ We did not study diurnal variation in gut and pancreatic hormones although all visits took place in the morning, and for insulin, glucagon, GLP-1, PYY and GIP, diurnal changes are likely to be influenced primarily by nutrient ingestion at mealtimes.^7^ Although hormone concentrations can be influenced by age, gender and obesity, the current study has inadequate sample size to provide formal reference intervals or partitioned reference estimations for subgroups of the population.^13^

Despite its limitations, this study provides a comprehensive set of data for expected concentrations of gut hormones in healthy human subjects. Our data show reasonable consistency with other published work, although direct comparison is difficult due to the variety of stimulation tests and analytical methods used. For example, Vilsbøll et al. studied fasting and postprandial gut hormone responses in eight healthy individuals who had previously had a normal OGTT.^23^ A 260 kcal meal of bread, margarine and jam was given with milk. For GLP-1, fasting concentrations of 15–20 pmol/L (50–66 pg/mL) were obtained and concentrations peaked at 30 pmol/L (99 pg/mL) after the meal (1 pmol/L GLP-1 = 3.297 pg/mL GLP-1). For GIP, fasting concentrations were around 15 pmol/L (66 pg/mL) with a peak concentration of 80–90 pm (352–396 pg/mL) after the meal (1 pmol/L GIP = 4.4 pg/mL GIP). Visbǿll’s population had lower concentrations of fasting and postprandial insulin (fasting 15–20 pmol/L; peak ∼180 pmol/L) compared with the population under investigation in the current study, but participants had been prescreened with an OGTT prior to enrolment and individuals with a family history of diabetes were excluded. Concentrations of fasting C-peptide in Visbǿll’s study were 400 pmol/L with peak concentrations of 1500 pmol/L after the meal.^23^ In contrast, Alsalim et al. studied 24 healthy lean volunteers who had a meal of 511 kcal, consisting of a sirloin steak with potatoes, vegetables and sorbet.^24^ Concentrations of intact GIP were 10 pmol/L (44 pg/mL) fasting and peaked at around 75 pmol/L (330 pg/mL) postprandially. Insulin concentrations were around 30 pmol/L fasting and around 250 pmol/L postprandially. C-peptide concentrations were 300 pmol/L fasting and around 1000 pmol/L postprandially. Unfortunately, concentrations of total GLP-1 and GIP were not reported.^24^ In the current study, the use of a liquid meal, rather than solid food, is likely to explain the more rapid increase in gut hormones seen in comparison to Visbǿll and Alsalim’s work.^23,24^

In the current study, glucagon concentrations after the SMT generally exhibited a small transient elevation, and thereafter remained similar to baseline. This likely reflects a complex balance between inhibitory signals acting on pancreatic alpha cells, including glucose and GLP-1, and stimulatory signals such as amino acids. After the OGTT, we expected to see a less complex picture, as glucagon should be predominantly suppressed by the elevated glucose and GLP-1 concentrations. Indeed, in the majority of healthy volunteers, post-OGTT glucagon concentrations reached a nadir by 45 to 60 min. A few participants (6/28), however, exhibited an elevated glucagon of >50% at some point in the first hour. It is currently not clear whether this represents a true increase in biologically active glucagon from the pancreas, release of glucagon from the intestine, or detection by the glucagon assay of longer peptides containing the glucagon sequence that might be released from the gut or pancreas. It is also possible that under physiological conditions, the glucagon-stimulating effects of GIP outweigh the glucagon-suppressing effects of GLP-1.^25,26^ It has been proposed that the gut can release glucagon under certain conditions, including following total pancreatectomy where pancreatic release should be impossible,^27^ or after Roux-en-Y gastric bypass surgery.^28,29^ However, when GLP-1 secretion is high, some glucagon assays cross-react with longer proglucagon-derived species containing the glucagon sequence, particularly glicentin, leading to debate over whether the gut ever releases intact glucagon.^30,31^ In addition to these reports from populations with altered pathology, and although we were unable to find active glucagon in human intestinal tissue by LC-MS,^32^ our data suggest that some healthy people may also exhibit post-OGTT glucagon elevation.

In conclusion, this comprehensive set of expected values for fasting and postprandial gastrointestinal and pancreatic hormone concentrations in healthy human subjects facilitates future comparison between healthy individuals and those with metabolic or endocrine disorders in response to an OGTT or standardized liquid meal.

## Supplemental Material

sj-pdf-1-acb-10.1177_0004563220975658 - Supplemental material for Expected values for gastrointestinal and pancreatic hormone concentrations in healthy volunteers in the fasting and postprandial state Supplemental material, sj-pdf-1-acb-10.1177_0004563220975658 for Expected values for gastrointestinal and pancreatic hormone concentrations in healthy volunteers in the fasting and postprandial state by Claire L Meek, Hannah B Lewis, Keith Burling, Frank Reimann and Fiona Gribble in Annals of Clinical Biochemistry
