# Supplementary material for: Expected values for gastrointestinal and pancreatic hormone concentrations in healthy volunteers in the fasting and postprandial state
Source: Ann Clin Biochem. 2020 Dec 7;58(2):108–16. doi: 10.1177/0004563220975658 (PMC7961662; doi:10.1177/0004563220975658)
Supplement: sj-pdf-1-acb-10.1177_0004563220975658 - Supplemental material for Expected values for gastrointestinal and pancreatic hormone concentrations in healthy volunteers in the fasting and postprandial state [file sj-pdf-1-acb-10.1177_0004563220975658.pdf]

Supplementary Material

Further Details on Analytical Methodology

All blood analyses were performed in the Department of Clinical Biochemistry in Addenbrooke's Hospital in the Core Biochemistry Assay Laboratory, which is routinely used for diagnostic clinical purposes. During the period of this study, the laboratory was accredited by the UK Accreditation service (UKAS) under Clinical Pathology Accreditation (CPA) (UK) and later under ISO-15189. Detailed analysis of the methodology is outlined below.

For biochemistry testing, serum samples were taken and allowed to clot for 10 minutes after venesection. Samples were centrifuged at 3500 g for ten minutes at 4°C, separated and frozen at -80°C until analysis. Alanine aminotransferase (ALT) and creatinine were measured using a Siemens' Dimension analyser with CVs of <2% within the reference range. Thyroid stimulating hormone (TSH) was measured using a Bayer ADVIA Centaur immunoassay system with CVs of <6% within the reference range. HbA1c (aligned to the International Federation of Clinical Chemistry (IFCC) method) was measured on fresh whole plasma via high performance liquid chromatography (HPLC) using a Tosoh analyser (HLC-723G8) with CVs of <5% within the reference range. Glucose concentrations were measured in plasma using a hexokinase method on the Siemens' Dimension analyser with CVs of <2% within the reference range.

For analysis of total GLP-1, GIP and PYY, samples were taken into EDTA plasma tubes, placed on ice immediately after venesection and centrifuged at 3500g at 4°C for ten minutes. The sample was aliquotted and frozen at -80°C prior to batch analysis which was done in duplicate. GLP-1 was measured using the Mesoscale Discovery Total GLP-1 kit, which employs a sandwich immunoassay with an electrochemiluminescent detection method. This assay claims to measure all endogenous forms of GLP-1 (including GLP-$1_{1\text{-}36}$, GLP-$1_{1\text{-}37}$, GLP-$1_{7\text{-}36}$, GLP-$1_{7\text{-}37}$, GLP-$1_{9\text{-}36}$, and GLP-$1_{9\text{-}37}$) and uses a synthetic standard. This method has a range of 1.4-1000 pg/ml and coefficients of variation (CVs) of 5.2-8.2% for most of the analytical range. As expected, CVs at the lower end of the analytical range were less robust (15.4% at 5.5 pg/ml).

Total GIP and total PYY were measured in duplicate using Mesoscale Discovery immunoassay kits. Both immunoassays use a synthetic standard. For GIP, this method has a range of 1.0-2500 pg/ml and inter-assay CVs of 9.3-11.0% within the physiological range. The assay measures total GIP including $GIP_{1\text{-}42}$ and $GIP_{3\text{-}42}$. The PYY immunoassay measures both $PYY_{1\text{-}36}$ and $PYY_{3\text{-}36}$ and has a range of 30-3000 pg/ml. Inter-assay CVs of 7.8-16.4% were obtained within the physiological range.

For measurement of insulin, C-peptide and proinsulin, samples were taken into lithium heparin plasma tubes, placed on ice immediately after venesection and centrifuged at 3500g at 4°C for ten minutes. After centrifugation, samples were aliquotted and frozen at -80°C prior to duplicate analysis in batches. Analysis of insulin was performed using the Diasorin Liaison which is a sandwich immunoassay with a chemiluminescent detection method using isoluminol conjugated to the secondary antibody. For insulin quantitation, this method has a range of 3-3000 pmol/l and intra-assay CVs of 5.0-6.0% across the analytical range. The insulin

standard is aligned with the World Health Organisation (WHO) first international reference preparation 66/304. Insulin, c-peptide and glucose assays used third party QC's from Bio-Rad (Lyphochek Immunoassay plus a low level in-house (based on the BioRad material to have clinically relevant ranges) with Multiqual).

This assay is known to have low levels of cross-reactivity to other related molecules, as shown in the table below. This information was provided by the manufacturer and was assessed in accordance with the guidelines of Clinical and Laboratory Standards Institute (CLSI, USA), Document No. EP07-A2.

**Compound**		**% Cross- reactivity**
Human C-Peptide	200 ng/mL	-1.4
Human proinsulin	200 ng/mL	-0.9
Human glucagon	200 ng/mL	-0.8
Insulin-like growth factor I (IGF-I)	200 ng/mL	2.9
Bovine insulin	0.5 ng/mL	74.7
Porcine insulin	0.5 ng/mL	191.3

Glucagon was measured in singulate using the Mercodia sandwich immunoassay kit which is reported to use two monoclonal antibodies directed to antigens at each end of the glucagon molecule to provide specificity for the measurement of glucagon. This is particularly important with products of the proglucagon gene where multiple forms can exist due to different post translational modification in different tissues, which may all be present in plasma. This method gives good specificity with minimal interference from related peptides (4.4% oxyntomodulin, 0.8% glicentin, <0.3% GLP-1 and GLP-2, <0.1% mini-glucagon). The Mercodia method has a range of 5-1000 pmol/l and inter-assay CVs of 8-10% were achieved for most of the assay range. As expected, CVs were less robust (~21.2%) near the lower limit of detection (LLOD). The Mercodia glucagon assay uses a synthetic standard which is calibrated against the WHO first International reference preparation 69/194.

For measurement of C-peptide and intact proinsulin, samples were taken into lithium heparin plasma tubes, placed on ice immediately after venesection and centrifuged at 4000 rpm at 4°C for ten minutes, sample aliquotted and frozen at -80°C prior to duplicate analysis in batches. Analysis of C-peptide was performed using the Diasorin Liaison which is a sandwich immunoassay with a chemiluminescent detection method using isoluminol conjugated to the secondary antibody. For C-peptide quantitation, this method has a range of 9-9900 pmol/l and intra-assay CVs of 4.6-7.6 % across the analytical range. The C-peptide standard is aligned with the WHO first international reference preparation 84/510. The C-peptide assay also used third party QC's from Bio-Rad (Lyphochek Immunoassay plus a low level in-house (based on the BioRad material to have clinically relevant ranges) with Multiqual).

Intact Proinsulin was measured using an in-house, time-resolved, fluorometric assay on an Auto-DELFIA immunoassay system using antibodies and other reagents from Perkin-Elmer life Sciences. Intact proinsulin standard was supplied by the National Institute for Biological Standards and Controls (WHO First international

reference preparation 84/611). All QC are prepared in batched and stored at -70°C. This method uses two monoclonal antibodies, the second of which is labelled with Europium to allow detection. The assay range is 1.25-100 pmol/l and inter-assay CVs of 4.9-11.8% were achieved throughout the analytical range. Thorough testing with sources of common analytical interference has not been performed for this assay, but it performs adequately in samples from patients with moderate amounts of bilirubin, triglycerides and haemolysis. There is 100% cross reactivity of proinsulin in the Diasorin Liaison XL C-peptide CLIA as demonstrated in a previous study and as reflected in the manufacturer's instructions which were updated based on the data from this study (Cheah et al, 2018. Refractory spontaneous hypoglycaemia: a diagnostic conundrum. Endocrinology, Diabetes & Metabolism).

Non-esterified free fatty acids (NEFA; FFA) were measured on rapidly processed serum samples using a Roche enzymatic colorimetric assay according to manufacturer's instructions. The range of this assay is 50-1500 umol/l and CVs of 5.0-12.3% were achieved within the physiological range. The standard is a synthetic preparation (Biorad lyphochek) which is used in dilution.

Supplementary Table 1: Lower range limit (LRL; 2.5th percentile), median and upper range limit (URL; 97.5th percentile) for gastrointestinal and pancreatic peptides before and after a 75g OGTT. Note that intact proinsulin and C-peptide were measured at timepoints 0, 30, 60, 90, 120, 180 and 240 minutes only on samples from 20 participants. The remaining analytes were measured on samples from 28 participants.

		Fasting	+15 min	+30 min	+45 min	+60 min	+90 min	+120 min	+150 min	+180 min	+210 min	+240 min
Glucose mmol/l	LRL	3.7	5.2	5.6	3.7	3.9	3.9	2.3	3.0	3.3	3.3	3.3
	Median	5.2	6.9	7.6	7.3	7.1	5.9	5.7	5.4	4.5	4.2	4.4
	URL	6.4	9.5	11.3	12.0	11.8	9.2	8.6	8.6	8.4	5.9	5.8
Insulin pmol/l	LRL	9.0	24.8	103	112	95.8	62.4	26.5	12.5	6.0	3.6	5.6
	Median	33.8	220	343	342	355	284	172	133	40.1	34.9	26.8
	URL	180	1042	1779	1545	1541	1635	1180	1499	727	170	105
Proinsulin pmol/l	LRL	1.3	NA	2.7	NA	4.5	5.1	3.8	NA	2.2	NA	1.6
	Median	3.0	NA	6.7	NA	10.1	12.1	13.9	NA	7.5	NA	4.7
	URL	5.3	NA	29.3	NA	27.2	29.4	32.2	NA	22.0	NA	11.3
C-peptide pmol/l	LRL	235	NA	614	NA	1070	998	715	NA	371	NA	252
	Median	510	NA	1900	NA	2230	2170	1790	NA	795	NA	454
	URL	805	NA	3360	NA	4040	3220	3320	NA	2290	NA	1110
Glucagon pmol/l	LRL	6.6	6.4	5.2	5.0	5.0	5.0	4.7	5.0	5.0	5.0	5.0
	Median	25.1	15.7	15.7	10.2	9.6	9.8	10.0	9.2	11.4	18.6	21.0
	URL	61.1	56.0	63.1	62.3	78.3	40.7	49.4	48.1	62.3	51.7	55.1
GLP-1 pg/ml	LRL	3.0	7.5	6.3	6.1	5.4	3.7	2.9	3.5	3.4	2.2	2.8
	Median	9.3	20.2	26.7	15.8	14.2	15.5	11.7	9.3	7.2	8.5	7.0
	URL	33.5	80.2	70.8	79.8	90.2	29.5	29.3	16.2	18.8	18.5	26.8
PYY pg/ml	LRL	27.3	31.2	45.9	38.6	38.6	38.8	30.0	30.0	30.0	27.4	30.0
	Median	59.1	76.8	81.3	81.9	74.3	67.9	59.3	53.5	51.2	46.1	46.2
	URL	123	181	180	189	205	140	136	135	148	140	164
GIP pg/ml	LRL	12.4	40.4	44.3	39.7	59.3	67.4	44.7	20.6	17.6	14.6	17.6
	Median	29.4	118	125	126	126	126	103	95.7	62.5	39.4	28.6
	URL	76.9	503	434	391	230	252	362	298	228	231	90.9
FFA umol/l	LRL	74.4	74.0	54.0	39.5	25.2	21.7	16.9	15.6	25.0	25.0	50.0
	Median	344	308	200	88.9	65.3	50.0	50.0	50.0	69.0	216	407
	URL	793	969	474	290	252	134	166	710	668	935	1006

Abbreviations: OGTT: oral glucose tolerance test, FFA: free fatty acids, GLP-1: glucagon-like peptide-1, GIP: glucose-dependent insulinotropic polypeptide, LRL: lower range limit; 2.5th percentile, PYY: peptide YY, URL: upper range limit; 97.5th percentile.

Supplementary Table 2: Lower range limit (LRL; 2.5th percentile), median and upper range limit (URL; 97.5th percentile) for gastrointestinal and pancreatic hormones before and after a standardised meal, based on samples taken from 28 participants.

		Fasting	+15 min	+30 min	+45 min	+60 min	+90 min	+120 min	+150 min	+180 min	+210 min	+240 min
Glucose mmol/l	LRL	4.5	5.0	4.6	3.7	2.8	2.8	3.4	3.7	4.2	4.2	4.3
	Median	5.3	6.2	6.3	6.2	5.0	5.0	5.1	5.0	5.0	5.0	5.1
	URL	6.3	7.4	9.2	8.6	7.9	7.9	6.4	6.1	6.0	5.6	5.6
Insulin pmol/l	LRL	7.2	66.1	99.9	143.7	121.5	32.0	11.0	7.8	4.1	3.8	4.8
	Median	46.5	163	371	363	250	143	104	43.7	35.8	25.5	24.0
	URL	174	749	1518	1331	1089	1030	647	297	122	106	86.4
Glucagon pmol/l	LRL	6.7	5.0	6.9	10.3	9.8	7.1	8.8	6.0	7.8	6.0	10.4
	Median	22.5	35.7	28.4	27.8	28.3	22.8	27.7	29.8	27.2	23.0	27.2
	URL	48.6	61.8	77.2	69.2	67.8	79.0	99.8	54.2	59.8	65.3	80.6
GLP-1 pg/ml	LRL	3.1	6.3	6.3	6.4	6.2	5.0	5.5	5.1	4.4	5.0	4.0
	Median	9.6	19.4	20.3	15.9	14.8	15.4	15.6	14.8	14.7	11.6	10.8
	URL	17.2	58.1	41.2	37.9	37.9	35.4	41.3	25.9	23.3	22.9	25.6
PYY pg/ml	LRL	29.2	38.4	43.8	39.4	34.1	42.8	42.3	41.4	38.9	31.0	30.0
	Median	58.9	72.2	86.5	79.2	67.6	71.9	74.8	75.3	66.9	69.9	61.8
	URL	165	187	173	166	150	154	143	149	139	122	114
GIP pg/ml	LRL	11.3	26.7	80.6	101	105	80.9	41.1	36.3	22.6	18.1	16.9
	Median	25.1	175	253	158	234	211	154	101	63.7	49.5	39.8
	URL	127	527	828	613	623	558	417	442	407	193	90.6
FFA umol/l	LRL	102	79.2	43.6	37.8	35.5	39.5	42.6	34.4	73.1	76.7	73.2
	Median	364	277	166	105	81.4	65.8	81.3	116	178	321	435
	URL	1021	622	503	375	304	233	249	580	818	740	944

Abbreviations: FFA: free fatty acids, GLP-1: glucagon-like peptide-1, GIP: glucose-dependent insulinotropic polypeptide, LRL: lower range limit; 2.5th percentile, PYY: peptide YY, URL: upper range limit; 97.5th percentile.

Supplementary Table 3: Bootstrapped lower range limit (LRL; 2.5th percentile), median and upper range limit (URL; 97.5th percentile) for gastrointestinal and pancreatic peptides before and after a 75g OGTT. Note that intact proinsulin and C-peptide were measured at timepoints 0, 30, 60, 90, 120, 180 and 240 minutes only on samples from 20 participants. The remaining analytes were measured on samples from 28 participants.

		Fasting	+15 min	+30 min	+45 min	+60 min	+90 min	+120 min	+150 min	+180 min	+210 min	+240 min
Glucose mmol/l	LRL	4.2	5.2	5.6	4.1	3.9	4.1	3.1	3.3	3.4	3.3	3.4
	Med	5.2	6.9	7.6	7.3	7.1	5.9	5.7	5.4	4.5	4.3	4.4
	URL	6.2	9.1	11.2	11.5	11.2	9.0	8.2	7.6	7.0	5.5	5.6
Insulin pmol/l	LRL	11.6	44.3	105.5	118.3	134.1	86.6	47.5	17.9	12.7	7.0	5.8
	Med	33.8	219.5	342.8	342.0	355.3	283.7	171.6	133.1	40.1	34.9	26.8
	URL	154.9	785.2	1,456.6	1,528.8	1,372.9	1,192.7	816.8	884.7	499.7	161.0	90.1
Proinsulin pmol/l	LRL	1.3	NA	2.8	NA	4.7	5.5	4.9	NA	2.7	NA	1.9
	Med	3.0	NA	6.7	NA	10.1	12.1	13.9	NA	7.5	NA	4.7
	URL	5.1	NA	23.8	NA	27.2	28.6	31.1	NA	21.9	NA	10.9
C-peptide pmol/l	LRL	241.2	NA	651.1	NA	1,160.3	1,043.9	774.4	NA	390.5	NA	257.2
	Med	510.0	NA	1,900.0	NA	2,230.0	2,170.0	1,790.0	NA	794.5	NA	454.0
	URL	781.3	NA	3,113.0	NA	3,569.8	3,175.0	3,139.5	NA	2,171.3	NA	1,038.3
Glucagon pmol/l	LRL	7.2	6.4	5.7	5.0	5.0	5.0	4.9	5.0	5.0	5.4	7.0
	Med	25.1	15.7	15.7	10.2	9.6	9.8	10.0	9.2	11.4	18.6	21.0
	URL	51.9	52.1	56.7	58.6	58.8	37.7	37.0	42.9	62.1	50.4	48.9
GLP-1 pg/ml	LRL	4.2	8.1	8.4	7.1	6.1	4.8	3.9	4.3	3.5	2.5	3.2
	Med	9.3	20.2	26.7	15.8	14.2	15.5	11.7	9.3	7.2	8.5	7.0
	URL	24.2	76.2	66.5	63.8	61.5	29.0	23.9	15.9	14.0	13.6	17.1
PYY pg/ml	LRL	32.8	37.1	48.4	50.8	45.5	39.3	35.4	36.7	32.4	29.1	30.8
	Med	59.1	76.8	81.3	81.9	74.3	67.9	59.3	53.5	51.2	46.1	46.2
	URL	101.8	167.6	179.3	166.2	170.6	127.4	108.7	102.7	100.9	94.3	109.0
GIP pg/ml	LRL	13.3	42.2	47.4	51.9	75.5	70.8	45.4	25.4	17.8	19.6	18.0
	Med	29.4	117.8	124.5	125.8	125.5	125.7	102.7	95.7	62.5	39.4	28.6
	URL	73.7	392.3	323.7	316.7	227.9	248.1	269.9	238.3	199.2	149.4	66.9
FFA umol/l	LRL	77.5	85.4	58.3	42.3	32.3	23.9	19.3	18.1	25.0	25.0	80.4
	Med	344.0	307.6	200.3	88.9	65.3	50.0	50.0	50.0	69.0	215.8	407.3
	URL	635.8	714.5	471.0	281.2	213.1	112.3	143.4	356.1	607.0	811.9	956.9

Abbreviations: OGTT: oral glucose tolerance test, FFA: free fatty acids, GLP-1: glucagon-like peptide-1, GIP: glucose-dependent insulinotropic polypeptide, LRL: lower range limit; 2.5th percentile, PYY: peptide YY, URL: upper range limit; 97.5th percentile.

Supplementary Table 4: Bootstrapped **l**ower range limit (LRL; 2.5th percentile), median and upper range limit (URL; 97.5th percentile) for gastrointestinal and pancreatic hormones before and after a standardised meal, based on samples taken from 28 participants.

		Fasting	+15 min	+30 min	+45 min	+60 min	+90 min	+120 min	+150 min	+180 min	+210 min	+240 min
Glucose mmol/l	LRL	4.7	5.1	4.9	3.9	3.4	3.2	3.7	4.2	4.2	4.3	4.3
	Med	5.3	6.2	6.3	6.2	5.0	5.0	5.1	5.0	5.0	5.0	5.1
	URL	6.2	7.3	8.7	8.4	7.6	7.4	6.2	6.0	5.7	5.6	5.5
Insulin pmol/l	LRL	17.4	68.9	111.3	147.3	129.1	32.5	16.2	9.8	6.9	6.7	5.9
	Med	46.5	162.9	371.2	362.5	250.0	143.0	103.7	43.7	35.8	25.5	24.0
	URL	148.7	683.0	1,216.4	1,126.5	821.2	692.0	432.7	248.4	105.6	91.2	78.6
Glucagon pmol/l	LRL	10.2	11.3	12.6	12.9	11.9	7.9	10.6	10.3	11.6	9.1	10.8
	Med	29.6	35.7	28.4	27.8	28.3	31.1	27.7	29.8	27.2	23.0	27.2
	URL	54.9	58.5	74.7	57.8	61.0	77.2	72.3	50.5	54.9	53.5	63.9
GLP-1 pg/ml	LRL	3.5	7.3	7.9	6.8	6.9	6.1	6.6	6.1	6.0	5.1	4.2
	Med	9.6	19.4	20.3	15.9	14.8	15.4	15.6	14.8	14.7	11.6	10.8
	URL	16.8	45.1	40.6	39.1	30.3	28.7	29.9	24.8	22.9	19.5	21.5
PYY pg/ml	LRL	32.4	40.7	44.1	47.1	39.4	43.7	44.5	44.4	39.5	39.7	32.3
	Med	58.9	72.2	86.5	79.2	67.6	71.9	74.8	75.3	66.9	69.9	61.8
	URL	124.4	180.9	172.9	156.4	130.4	128.4	142.3	130.5	114.5	101.2	105.4
GIP pg/ml	LRL	13.1	42.0	98.6	115.2	112.2	86.2	51.2	37.5	27.7	19.7	18.2
	Med	25.1	174.7	252.7	257.6	234.3	210.9	154.1	100.8	63.7	49.5	39.8
	URL	81.3	412.9	675.5	539.0	493.7	481.5	373.3	418.9	281.0	137.2	89.6
FFA umol/l	LRL	122.4	83.2	48.9	41.3	40.5	43.6	42.6	44.5	75.9	92.0	129.0
	Med	390.2	284.0	172.6	105.7	81.2	63.2	77.3	114.0	174.4	305.9	446.4
	URL	1,020.5	546.1	457.8	368.5	301.3	194.8	222.3	412.8	657.1	689.5	930.4

Abbreviations: FFA: free fatty acids, GLP-1: glucagon-like peptide-1, GIP: glucose-dependent insulinotropic polypeptide, LRL: lower range limit; 2.5th percentile, PYY: peptide YY, URL: upper range limit; 97.5th percentile.

Supplementary Table 5: Assay product code and supplier

Analyte	Instrument/kit	Product code	Supplied by
**Glucose**	Siemens' Dimension	Product code DF40; part number 10444971	Siemens
**Insulin**	DiaSorin Liaison	310360	DiaSorin
**Total GLP-1**	Mesoscale Discovery Total GLP-1 ver. 2 kit	K150JVC-4	MSD
**PYY**	Mesoscale Discovery Total PYY kit	K151MPD-2	MSD
**GIP**	Mesoscale Discovery Total GIP kit	K151RPD-4	MSD
**FFA**	Roche FFA kit	11383175001	Sigma Aldrich (now Merck)
**Glucagon**	Mercodia Glucagon kit	10-1271-01	Mercodia
**C-peptide**	DiaSorin Liaison	316171	DiaSorin
**Proinsulin**	In-house assay anlysed on PerkinElmer AutoDELFIA	In-House	n/a
